# Supplementary material for: Wings and halteres act as coupled dual oscillators in flies
Source: eLife. 2021 Nov 16;10:e53824. doi: 10.7554/eLife.53824 (PMC8629423; doi:10.7554/eLife.53824)
Supplement: Supplementary file 1. — (A) Table of p-values for a one-sided Wilcoxon signed-rank sum test at all wing length bins for epimeral ridge cut flies in Figure 3A. (B) Table of p-values for a one-sided Wilcoxon signed-rank sum test between haltere and wing pairs at all haltere loads in Figure 5. [file elife-53824-supp1.docx]

Supplementary file 1A: p values for a one-sided Wilcoxon signed rank sum test at all wing length bins for epimeral ridge cut flies in Figure 3A.

| Wing length bin | Epimeral Ridge Cut  Haltere -wing | Control Haltere – wing |
| --- | --- | --- |
| (0.9, 1.0) | 0.962632 | 0.553631 |
| (0.7, 0.9) | 0.458256 | 0.541744 |
| (0.6, 0.7) | 0.039808 | 0.657085 |
| (0.5, 0.6) | 0.039808 | 0.250092 |
| (0.4, 0.5) | 0.021557 | 0.021557 |
| (0.3, 0.4) | 0.021557 | 0.021557 |

Supplementary file 1B: p values for a one-sided Wilcoxon signed rank sum test between haltere and wing pairs at all haltere loads in Figure 5.

|  | Loaded Haltere - wing | Control Haltere - wing |
| --- | --- | --- |
| Intact haltere | 0.875568 | 0.827384 |
| Load 1 | 0.963031 | 0.986146 |
| Load 2 | 0.013854 | 0.662929 |
| Load 3 | 0.013854 | 0.986146 |
| Load removed | 0.231536 | 0.342915 |
